# Efficacy and safety of PD1/PDL1 inhibitors combined with radiotherapy and anti-angiogenic therapy for solid tumors: A systematic review and meta-analysis

**DOI:** 10.1097/MD.0000000000033204

**Published:** 2023-03-10

**Authors:** Feng Xian, Jing Wu, Liming Zhong, Guohui Xu

**Affiliations:** a Department of Oncology, Nanchong Central Hospital, The Second Clinical Institute of North Sichuan Medical College, Nanchong, China; b School of Medicine, University of Electronic Science and Technology of China, Chengdu, China; c Department of Operations Management, Nanchong Central Hospital, The Second Clinical Institute of North Sichuan Medical College, Nanchong, China.

**Keywords:** anti-angiogenic drug, efficacy, PD1/PDL1 inhibitors, radiotherapy, safety, solid tumors

## Abstract

The triple combination of programmed cell death 1 (PD1)/programmed cell death ligand 1 (PDL1) inhibitors, radiotherapy (RT), and anti-angiogenesis agents has been widely used in the treatment of solid tumors and has shown positive efficacy. We conducted a meta-analysis to evaluate the efficacy and safety of PD1/PDL1 inhibitors combined with anti-angiogenic agents and RT for the treatment of solid cancers.

**Methods:**

A systematic search of PubMed, Embase, Cochrane Library, and Web of Science databases was conducted from inception to October 31, 2022. Studies involving patients with solid cancers who received PD1/PDL1 inhibitors combined with RT and anti-angiogenic agents treatment that reported overall response rate, complete remission rate, disease control rate, and adverse events (AEs) were included. A random-effects or fixed-effects model was used for the pooled rates, and 95% confidence intervals (CIs) were determined for all outcomes. The quality of the included literature was assessed using the methodological index for nonrandomized studies critical appraisal checklist. Egger test was used to assess the publication bias in the included studies.

**Results:**

Ten studies (4 nonrandomized controlled trials and 6 single-arm trials), including 365 patients, were identified and included in the meta-analysis. The pooled overall response rate after treatment with PD1/PDL1 inhibitors combined with RT and anti-angiogenic agents was 59% (95% CI: 48–70%), whereas the disease control rate and complete remission rate were 92% (95% CI: 81–103%) and 48% (95% CI: 35–61%), respectively. Moreover, the meta-analysis showed that compared with triple-regimen, monotherapy or dual-combination treatment did not improve overall survival (hazard ratio = 0.499, 95% CI: 0.399–0.734) and progression-free survival (hazard ratio = 0.522, 95% CI: 0.352–0.774). The pooled rate of grade 3 to 4 AEs was 26.9% (95% CI: 7.8%–45.9), and the common AEs to triple therapy included leukopenia (25%), thrombocytopenia (23.8%), fatigue (23.2%), gastrointestinal discomfort (22%), increased alanine aminotransferase (22%), and neutropenia (21.4%).

**Conclusion:**

In the treatment of solid tumors, PD1/PDL1 inhibitors combined with RT and anti-angiogenic drugs achieved a positive response and better survival benefits than monotherapy or dual therapy. In addition, combination therapy is tolerable and safe.

**Registration:**

PROSPERO ID: CRD42022371433.

## 1. Introduction

Programmed cell death 1 (PD1) and programmed cell death ligand 1 (PD-L1) have been demonstrated as significant cancer therapeutic targets because they play an important role in facilitating immune evasion.^[1]^ Currently, patients with various cancers can achieve long-lasting survival benefits from anti-PD1/PDL1 treatment, which has revolutionized medical oncology.^[2]^ PD1 inhibitors (nivolumab, pembrolizumab, camrelizumab, and sintilimab) and PD-L1 inhibitors (durvalumab and atezolizumab) have been approved for the treatment of solid tumors in different clinical trials, including non-small-cell lung cancer (NSCLC), hepatocellular carcinoma (HCC), high-grade gliomas, nasopharyngeal carcinoma (NPC), and melanoma.

Although most patients with solid cancers benefit from anti-PD1/PD-L1 therapy, the general response rate remains unsatisfactory. Evidence suggests that targeting both tumor vessels and immune cells could increase their effectiveness in cancer patients.^[3]^ Studies have confirmed that the combination of anti-angiogenesis therapy and anti-PD1/PDL1 develops a positive reinforcing feedback loop to normalize tumor blood vessels, relieve hypoxia via increased tumor perfusion, and enhance the activation and infiltration of effector T cells, thus providing survival benefits.^[4]^ Currently, the National Comprehensive Cancer Network recommends combination therapy as a treatment for multiple solid cancers. Although the dual combination can improve patient outcomes for certain types of tumors, approximately two-thirds of patients remain unresponsive.^[5]^

Therefore, the addition of radiotherapy (RT) could further enhance the antitumor efficacy of the dual-combination therapeutic strategy of anti-PD1/PDL1 plus anti-angiogenesis, in part because of a direct effect on the tumor stroma, including immune cells and blood vessels.^[5]^ On the one hand, RT can reactivate immune response in the tumor microenvironment.^[6]^ On the other hand, angiogenesis can augment the efficacy of RT by normalizing tumor vessels and forming an immunology-friendly tumor microenvironment. Additionally, PD1/PDL1 inhibitors can overcome the upregulated inhibitory pathways and molecules triggered by RT, thereby enhancing the synergistic efficiency of RT.^[7]^

Although the combination therapy of anti-PD1/PDL1, RT, and anti-angiogenesis remains a therapeutic innovation to achieve promising clinical benefits, the clinical trials of the related combination have been reported in small population sizes and with specific series of adverse events (AEs) not fully defined, the exact efficacy and safety of the triple combination therapy in the treatment of solid cancer are still unclear. The purpose of this meta-analysis was to study the effectiveness and safety of anti-PD1/PDL1 combined with RT and anti-angiogenic agents in the treatment of solid tumors. The results of this study will provide guidance for clinical treatment.

## 2. Materials and Methods

### 2.1. Literature search strategy

This study was conducted following the Preferred Reporting Items for Systematic Reviews and Meta-Analysis guidelines (Supplementary Checklist S1, Supplemental Digital Content, http://links.lww.com/MD/I616).^[8,9]^ Two investigators independently searched for studies published before October 31, 2022 in PubMed, Embase, Cochrane Library, and Web of Science. The search keywords were “immune checkpoint inhibitors, ‘PD1 inhibitors’, ‘PDL1 inhibitors’, ‘nivolumab’, ‘pembrolizumab’, ‘camrelizumab’, and ‘radiotherapy’, ‘Stereotactic body radiation therapy’, ‘SBRT’, and ‘angiogenesis inhibitors’, ‘bevacizumab’, ‘apatinib’, ‘sorafenib’, and,” “cancer,” “carcinoma,” “carcinoma,” “tumor”; the search strategy for each database is shown in Supplementary Table S1, Supplemental Digital Content, http://links.lww.com/MD/I617. In addition, references to reviews and original studies were scanned to avoid missing studies that should be included.

### 2.2. Inclusion and exclusion criteria

The inclusion criteria were as follows: retrospective studies and prospective studies (including single-arm studies, cohort studies, and randomized control trials); patients who were pathologically diagnosed with any type of solid cancer; patients treated with PD1/PD-L1 inhibitors combined with anti-angiogenic drugs and RT; and studies that reported efficacy endpoints, including objective response rate (ORR), complete response rate (CRR), disease control rate (DCR), mortality rate (MR), and AEs.

The exclusion criteria were as follows: experiments performed in vitro or in vivo, but not based on patients; incomplete data for the targeted outcomes; patients with hematologic tumors, including leukemia, multiple myeloma, and malignant lymphoma; and studies published as conference abstracts, reviews, comments, case reports, and letters.

Two researchers independently reviewed the titles and abstracts of the studies and submitted eligible studies for full-text analysis to confirm whether they should be included in the meta-analysis. After each selection stage, the 2 researchers compared their findings. Inconsistencies were resolved and discussed by a third researcher.

### 2.3. Data extraction and definitions

Two researchers independently conducted data extraction, and any differences in opinion were resolved by participation with the third author in a joint discussion. The following data were extracted from each study: the first author’s name, year of publication, study design, median follow-up time, disease status, sample size, median age, treatment, and main outcomes. The main outcomes included ORR, DCR, and CRR according to the Response Evaluation Criteria in Solid Tumors (RECIST1.1).^[10]^ The following data were also extracted if the study contains: overall survival (OS), progression-free survival (PFS), and AEs. While the original survival data were hardly accessed, the extracted data from the Kaplan–Meier curves were obtained using the software Engauge Digitizer version 11.1 (Mark Mitchell 2019).

### 2.4. Quality assessment

The collected nonrandomized studies were evaluated using the nonrandomized study methodology (methodological index for nonrandomized studies).^[11]^ Single-arm trials with scores ≥ 8 were considered high-quality reports, whereas those with scores < 8 were considered low-quality reports. Nonrandom comparative studies with scores ≥ 13 were considered high-quality reports; otherwise, the studies were considered low-quality. All the included studies were assessed to have a low risk of bias.^[12]^ The results of the quality evaluation are presented in Table 1. As shown in Table 1, all the included studies were high-quality reports. Two independent reviewers completed the above tasks and discordance was resolved by consensus.

**Table 1:** Basic characteristics of included studies.

Study	Year	Country	Caner type	Trail design	Sample size	Median age (yr)	Interventions		Endpoints
							Experiment group	Control group	
Huang Y et al	2020	China	HCC	Single-arm	12	54.5	SBRT + sorafenib + camrelizumab + TACE	None	ORR, DCR, AEs
Hu L et al	2022	China	NPC	Single-arm	50	45	SBRT + apatinib + camrelizumab + chemotherapy	None	CRR, AEs
Liu Y et al	2021	China	RCC	Retrospective comparative	42	53	SBRT + anti-PD1 + TA	Anti-PD1 + TA	OS, ORR, DCR, CRR
Manzar GS et al	2022	USA	HCC	Single-arm	21	68	RT(2/3IMRT + 1/3underwent proton therapy) + atezolizumab + bevacizumab	None	ORR, DCR, AEs, MR
Sahebjam S et al	2021	USA	Gliomas	Single-arm	32	55.5	SBRT + pembrolizumab + bevacizumab	None	ORR, DCR, CRR, AEs
Su K et al	2022	China	HCC	Retrospective comparative	54	NR	IMRT + anti-PD1 + anti-angiogenesis	Anti-PD1 + anti-angiogenesis	OS, PFS, ORR, DCR, AEs, MR
Ye W et al	2022	China	NSCLC	Retrospective comparative	53	43.05	RT + apatinib + camrelizumab	RT + apatinib	ORR, DCR, CRR, AEs
Zhong L et al	2021	China	HCC	Single-arm	16	51.5	SBRT + anti-PD1/PDL1 + TA	None	ORR, DCR, CRR, AEs, MR
Zhang Z et al	2022	China	HCC	Retrospective comparative	30	52	SBRT + camrelizumab/tislelizummab + sorafenib + TACE	Sorafenib + TACE	OS, PFS, CRR, AEs
Shen J et al	2022	China	Solid tumors	Single-arm	55	NR	RT + camrelizumab + apatinib	None	ORR, DCR, AEs

AEs = adverse events, CRR = complete response rate, DCR = disease control rate, HCC = hepatocellular carcinoma, IMRT = intensity modulated radiotherapy, MR = mortality rate, NPC = nasopharyngeal carcinoma, NR = not reported, NSCLC = non-small-cell lung cancer, ORR = objective response rate, OS = overall survival, PD1 = programmed cell death 1, PFS = progression free survival, RCC = renal cell carcinoma, RT = radiotherapy, SBRT = stereotactic body radiotherapy, TA = targeted agents, TACE = transarterial chemoembolization.

### 2.5. Ethical consideration

The authors declare that they have obtained appropriate institutional review board approval or have followed the principles outlined in the Declaration of Helsinki for all human and animal experimental investigations. Informed consent was obtained from the participants involved in the investigations involving human subjects.

### 2.6. Statistical analysis

All data analyses were performed using STATA SE14.0 (StataCorp Station, TX). The pooled rates were analyzed using a random-effects model or a fixed-effect model. The pooled hazard ratios (HRs) were used to analyze the OS and PFS, and relative risks were used to analyze ORR, DCR, MR, and AEs, and all pooled effects were represented by the 95% confidence interval (CI). Cochran *Q* test and *I*^2^ statistics were used to assess the heterogeneity between studies, with a threshold of *P* < .1. The fixed-effects model was used for pooled results with low heterogeneity (*I*^2^ < 50%); otherwise, the random-effects model was used for the analysis. Sensitivity analysis was performed by excluding each study individually from the pooled results with high heterogeneity. Moreover, the symmetry of the visual observations of funnel plots and Egger test were used to assess publication bias. In addition, if the *P* value is not >0.05, the results above can be regarded as statistically significant.

## 3. Result

### 3.1. Study selection

**Figure 1.**
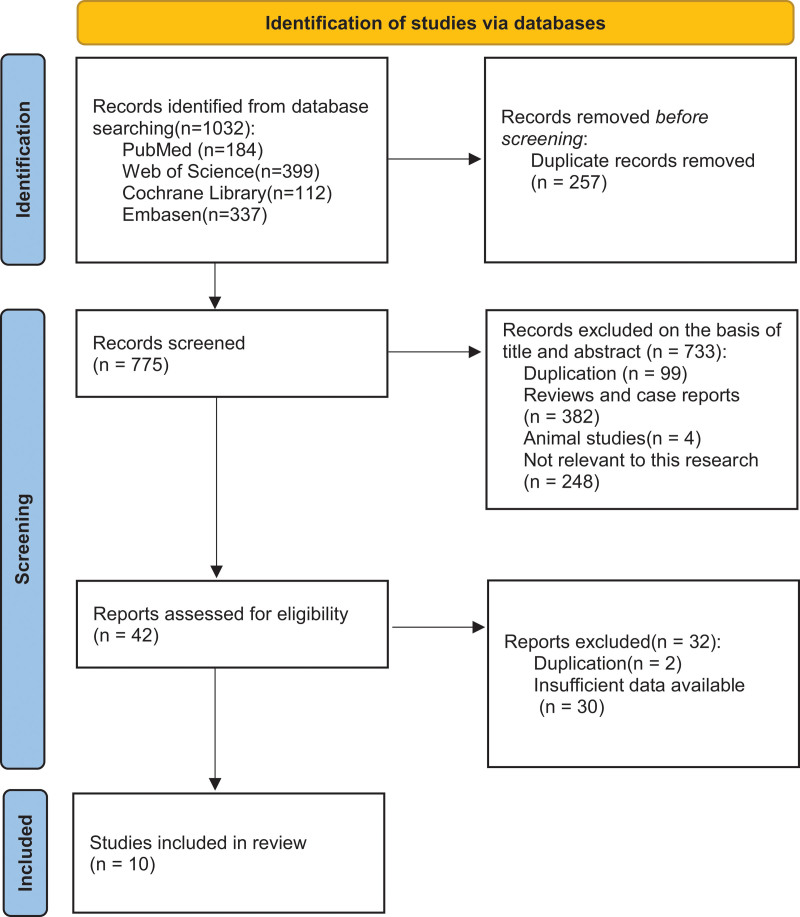
Flow diagram of study selection.

We searched the database for 1032 studies (184 in PubMed, 399 in Web of Science, 112 in Cochrane Library, and 337 in Embase). After eliminating duplicates (n = 257), titles and abstracts were browsed and filtered. The full text of the remaining 42 studies was screened, and 10 articles^[13–22]^ were finally included according to the inclusion criteria. The literature review and identification processes are shown in Figure 1.

### 3.2. Study characteristics

This meta-analysis included 10 studies, including 4 nonrandomized comparative studies and 6 single-arm trials, all published between 2020 and 2022. Among them, 5 studies evaluated HCC; one each evaluated NPC, renal cell cancer (RCC), NSCLC, and high-grade gliomas; and 1 study evaluated all solid tumors. The basic characteristics of the studies included in the meta-analysis are listed in Table 1.

### 3.3. Quality assessment

Four single-arm and 6 nonrandom comparative studies assessed using the methodological index for nonrandomized studies index score ranged from 11 to 13 points and 20 to 22 points, respectively, which were acceptable for the present meta-analysis (Table 2).

**Table 2:** Quality assessment of included studies.

Nonrandomized studies	(1)	(2)	(3)	(4)	(5)	(6)	(7)	(8)	(9)	(10)	(11)	(12)	Scores*
Huang Y et al	2	2	2	2	1	1	0	1	–	–	–	–	11 (full score: 16 points)
Hu L et al	2	2	2	2	1	0	2	0	–	–	–	–	11 (full score: 16 points)
Liu Y et al	2	2	2	2	1	2	2	0	2	2	1	2	20 (full score: 24 points)
Manzar GS et al	2	2	2	2	1	1	2	1	–	–	–	–	13 (full score: 16 points)
Sahebjam S et al	2	2	2	2	1	1	2	1	–	–	–	–	13 (full score: 16 points)
Su K et al	2	2	2	2	1	2	2	1	2	2	1	2	21 (full score: 24 points)
Ye W et al	2	2	2	2	1	1	2	1	2	2	1	2	20 (full score: 24 points)
Zhong L et al	2	2	2	2	1	1	2	0	–	–	–	–	13 (full score: 16 points)
Zhang Z et al	2	2	2	2	1	2	2	2	2	2	0	2	22 (full score: 24 points)
Shen J et al	2	2	2	2	1	1	1	0	–	–	–	–	11 (full score: 16 points)

Methodological items for nonrandomized studies: (1) A clearly stated aim: the question addressed should be precise and relevant in the light of available literature, (2) Inclusion of consecutive patients: all patients potentially fit for inclusion (satisfying the criteria for inclusion) have been included in the study during the study period (no exclusion or details about the reasons for exclusion), (3) Prospective collection of data: data were collected according to a protocol established before the beginning of the study, (4) Endpoints appropriate to the aim of the study: unambiguous explanation of the criteria used to evaluate the main outcome which should be in accordance with the question addressed by the study. Also, the endpoints should be assessed on an intention-to-treat basis, (5) Unbiased assessment of the study endpoint: blind evaluation of objective endpoints and double-blind evaluation of subjective endpoints, otherwise the reasons for not blinding should be stated, (6) Follow-up period appropriate to the aim of the study: the follow-up should be sufficiently long to allow the assessment of the main endpoint and possible adverse events, (7) Loss to follow-up <5%: all patients should be included in the follow-up. Otherwise, the proportion lost to follow-up should not exceed the proportion experiencing the major endpoint, (8) Prospective calculation of the study size: information of the size of detectable difference of interest with a calculation of 95% confidence interval, according to the expected incidence of the outcome event, and information about the level for statistical significance and estimates of power when comparing the outcomes; Additional criteria in the case of comparative study: (9) An adequate control group: having a gold standard diagnostic test or therapeutic intervention recognized as the optimal intervention according to the available published data, (10) Contemporary groups: control and the studied group should be managed during the same time period (no historical comparison), (11) Baseline equivalence of groups: the groups should be similar regarding the criteria other than the studied endpoints. Absence of confounding factors that could bias the interpretation of the results, (12) Adequate statistical analyses: whether the statistics were in accordance with the type of study with the calculation of confidence intervals or relative risk.

* The items are scored 0 (not reported), 1 (reported but inadequate), or 2 (reported and adequate); the global ideal score being 16 for non-comparative studies and 24 for comparative studies.

### 3.4. Efficacy

#### 3.4.1. Tumor response.

**Figure 2.**
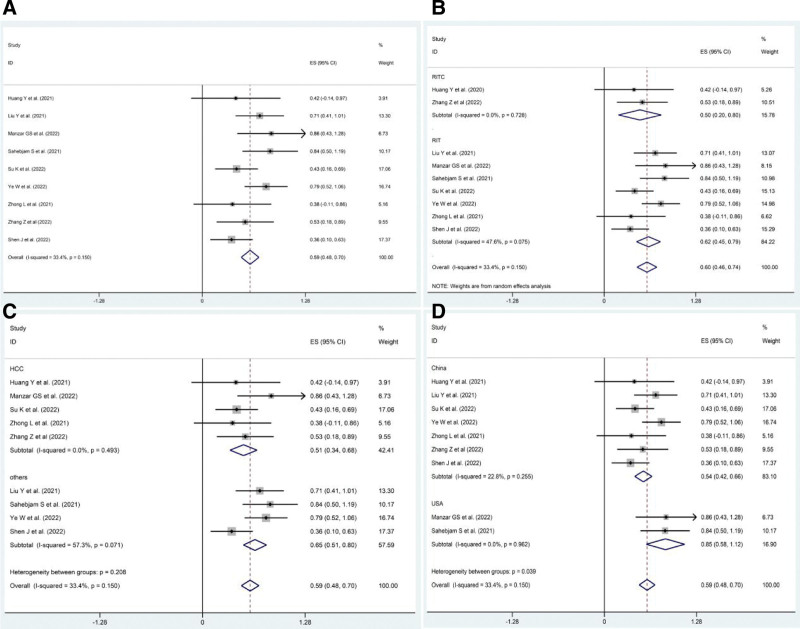
Pooled ORR in patients with solid cancers treated with RIT. (A) Pooled ORR in patients treated with RIT-based regimens. (B) Pooled ORR in patients treated with RIT plus chemotherapy (RITC) or not. (C) Pooled ORR in patients with different cancer: HCC group and others group. (D) Pooled ORR in patients from different countries: China group and USA group. ORR = overall response rate, RIT = PD1/PDL1 inhibitors combined with RT and anti-angiogenic agents.

**Figure 3.**
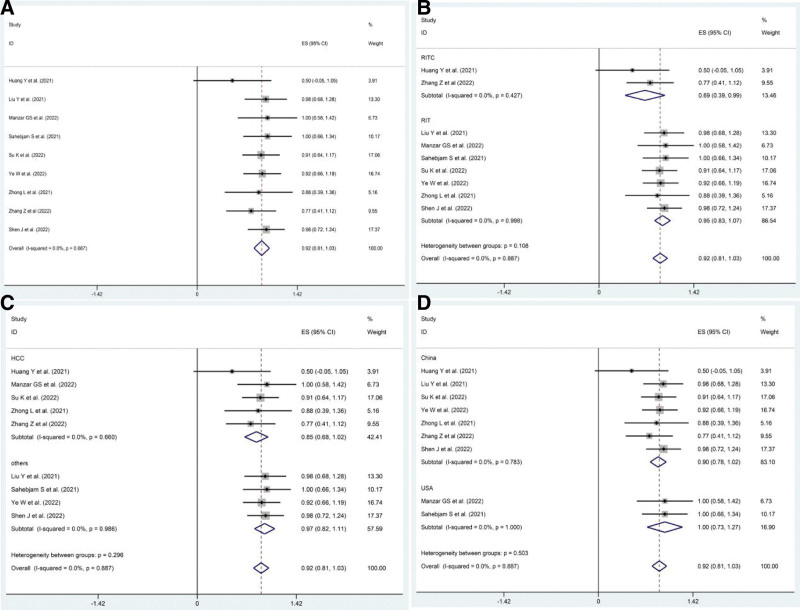
Pooled DCR in patients with solid cancers treated with RIT. (A) Pooled DCR in patients treated with RIT-based regimens. (B) Pooled DCR in patients treated with RIT plus chemotherapy (RITC) or not. (C) Pooled DCR in patients with different cancer: HCC group and others group. (D) Pooled DCR in patients from different countries: China group and USA group. DCR = disease control rate, RIT = PD1/PDL1 inhibitors combined with RT and anti-angiogenic agents, RITC = RIT plus chemotherapy.

Nine included studies^[13,15–22]^ reported ORR and DCR as clinical outcomes. The pooled ORR after PD1/PDL1 inhibitors combined with RT and anti-angiogenic agents (RIT) was 59% (95% CI: 48–70%, *P* = .00), whereas the pooled DCR was 92% (95% CI: 81–103%, *P* = .00) (Figs. 2A and 3A).

The included cases had various interference factors, including therapeutic regimens, countries, and types of cancer, which may have affected the pooled effects. This study included subgroup analyses. Among the different therapeutic regimens, RIT plus chemotherapy (RITC) therapy was administered in 2 studies,^[13,21]^ and the pooled ORR and DCR were 50% (95% CI: 20–80%, *P* = .001) and 69% (95% CI: 39–99%), respectively, while the pooled ORR and DCR were 61% (95% CI: 49–73%, *P* = .00) and 95% (95% CI: 83–107%) in the RIT therapy group. ORR in the RIT group was pooled using a random-effect model because of slight heterogeneity (*I*^2^ = 47.6%, *P* = .075) (Figs. 2B and 3B). Moreover, the pooled ORR in patients with HCC treated with RIT was 51% (95% CI: 34–68%, *P* = .00) and 65% (95% CI: 51–80%, *P* = .00) in patients without HCC, while the pooled DCR in HCC was 85% (95% CI: 68–102%, *P* = .00) and 97% (95% CI: 82–111%, *P* = .00) in other cancers (Figs. 2C and 3C). Furthermore, the pooled ORR and DCR in patients from the USA were 85% and 100% (*P* = .00), respectively, while the pooled ORR in patients from China was 54% (95% CI: 42–66%, *P* = .00), and the pooled DCR was 90% (95% CI: 78–102%, *P* = .00) (Figs. 2D and 3D).

**Figure 4.**
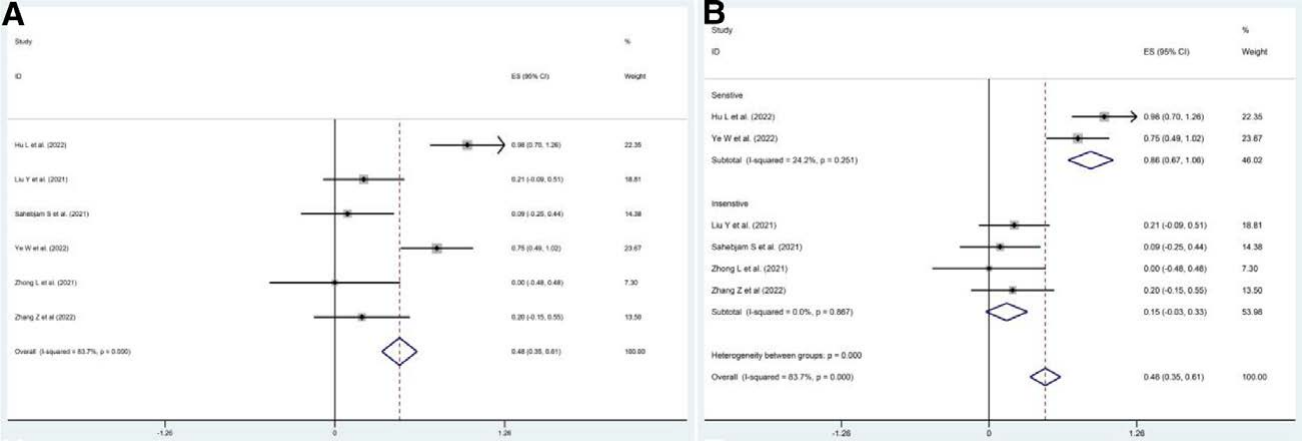
Pooled CRR in patients with solid cancers treated with RIT. (A) Pooled CRR in patients treated with RIT-based regimens. (B) Pooled CRR in patients with different sensitivity to RIT: sensitive group and insensitive group. CRR = complete remission rate, RIT = PD1/PDL1 inhibitors combined with RT and anti-angiogenic agents.

Six studies^[14,15,17,19–21]^ reported CRR. The pooled CRR was 48% (95% CI: 35–61%, *P* = .00), but there was significant heterogeneity among the 6 trials mentioned (*I*^2^ = 83.7%, *P* = .00) (Fig. 4A). Subgroup analysis, sensitivity analysis, and Galbraith plot confirmed the heterogeneity derived from the 2 studies (Hu et al^[14]^ and Sahebjam et al^[17]^) (Supplementary Figures S1–S3, Supplemental Digital Content, http://links.lww.com/MD/I618). The pooled CRR in the 2 trials was 86% (*P* = .00) and 15% (*P* = .098 > 0.05) in the other trials (Fig. 4B).

**Figure 5.**
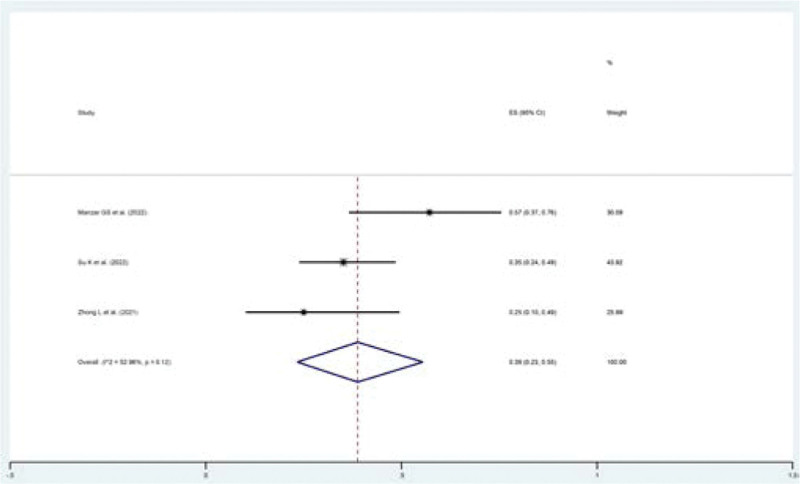
Pooled MR in patients with solid cancers treated with RIT. MR = mortality rate, RIT = PD1/PDL1 inhibitors combined with RT and anti-angiogenic agents.

MRs were investigated in 3 studies.^[16,18,20]^ The pooled MR was 39% (95% CI: 23–55%, *P* = .00), while the MR was combined with a random-effect model with minor heterogeneity (*I*^2^ = 52.96%, *P* = .12) (Fig. 5, Supplementary Figure S4, Supplemental Digital Content, http://links.lww.com/MD/I619).

**Figure 6.**
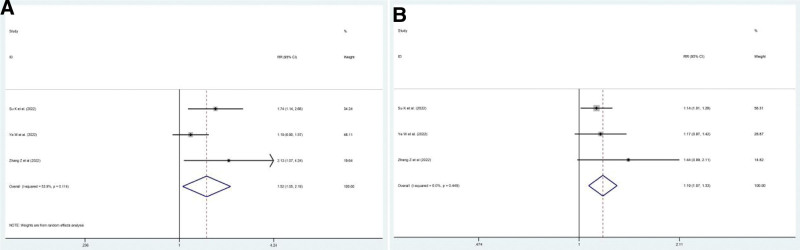
Pooled ORR and DCR in patients from nonrandom comparative trials. (A) Pooled ORR. (B) Pooled DCR. DCR = disease control rate, ORR = overall response rate.

Three nonrandom comparative studies^[18,19,21]^ have reported the ORR and DCR. The pooled ORR and pooled DCR were HR = 1.519 (95% CI: 1.052–2.194, *P* = .026) and HR = 1.194 (95% CI: 1.074–1.327), respectively (Fig. 6).

#### 3.4.2. Survival.

**Figure 7.**
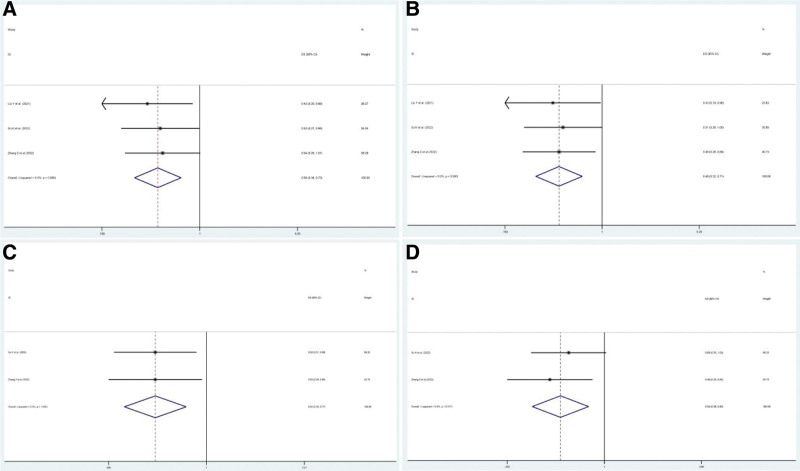
Pooled OS and PFS in patients from nonrandom comparative trials. (A) Pooled OS in univariate analysis. (B) Pooled OS in multivariable analysis. (C) Pooled PFS in univariate analysis. (D) Pooled PFS in multivariable analysis. OS = overall survival, PFS = progression-free survival.

Three studies^[15,18,21]^ reported OS, and 2 studies^[18,21]^ mentioned PFS. The relevant results showed that the OS of patients treated with monotherapy or double combined therapy was shorter than that treated with RIT/RITC (HR = 0.499, 95% CI: 0.399–0.734, *P* = .00; *I*^2^ = 0.0%, *P* = .868) in univariate analysis (Fig. 7A) and (HR = 0.477, 95% CI: 0.32–0.711) in multivariate analysis (Fig. 7B). The PFS in patients treated with monotherapy or double combined therapy was also shorter than treated with RIT/RITC (HR = 0.522, 95% CI: 0.352–0.774, *P* = .001; *I*^2^ = 0.0%, *P* = 1) in the univariate analysis (Fig. 7C) and (HR = 0.537, 95% CI: 0.36–0.801) in the multivariate analysis (Fig. 7D).

#### 3.4.3. AEs.

**Figure 8.**
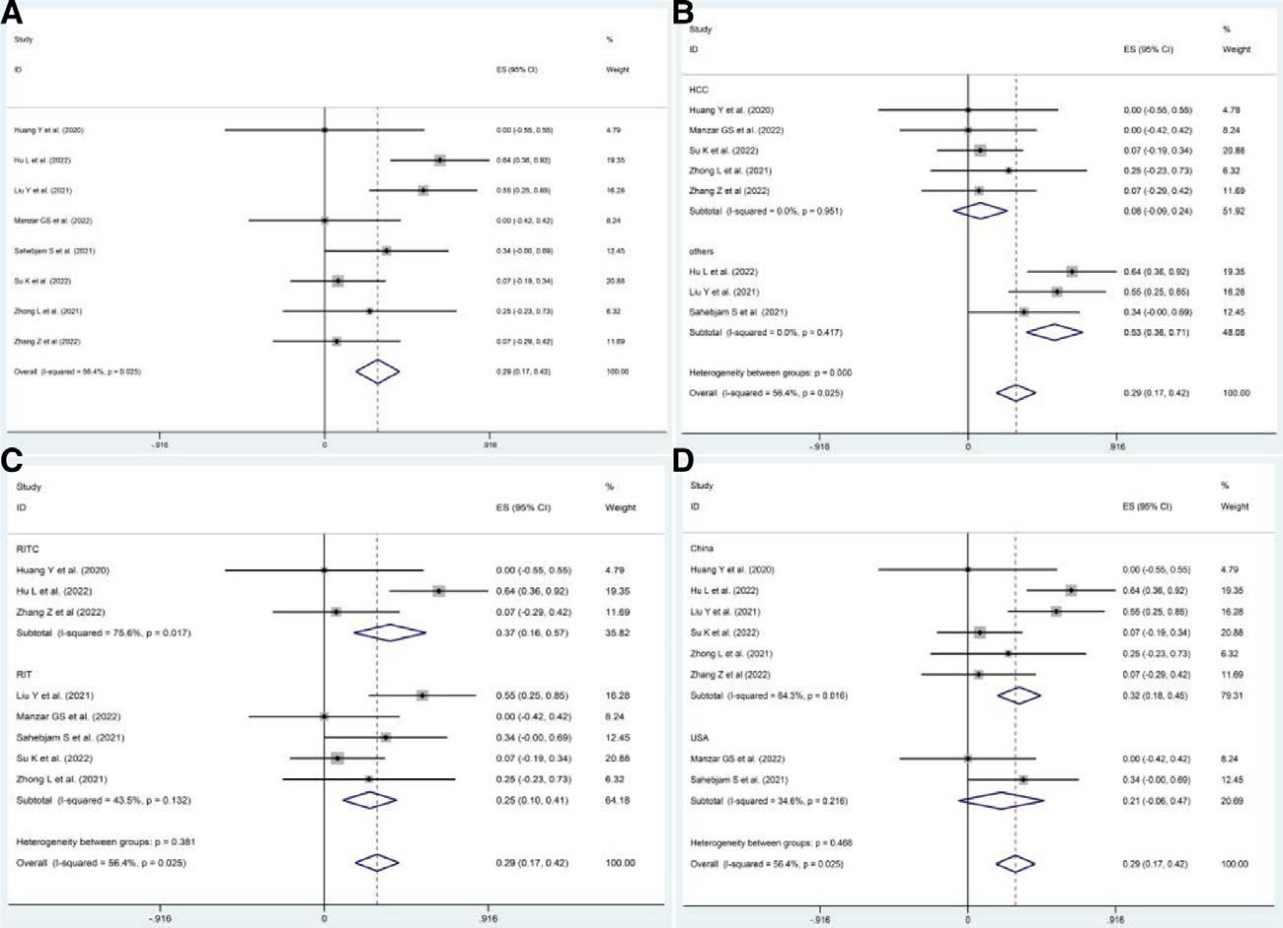
The pooled rates of grade 3 to 4 AEs in patients treated with RIT-base regimens. (A) Pooled rate of grade 3 to 4 AEs. (B) Pooled rate of grade 3 to 4 AEs in HCC group and other cancers group. (C) Pooled rate of grade 3 to 4 AEs in RITC group and RIT group. (D) Pooled rate of grade 3 to 4 AEs in China group and USA group. AEs = adverse events, RIT = PD1/PDL1 inhibitors combined with RT and anti-angiogenic agents, RITC = RIT plus chemotherapy.

AEs were reported in all the included studies. Three studies^[20–22]^ reported any-grade AEs and 8 studies^[13–18,20,21]^ reported grade 3 to 4 AEs. The pooled rate of any-grade AEs was 86% (95% CI: 77–93%, *P* = .00). Moreover, the pooled rate of grade 3 to 4 AEs was 26.9% (95% CI: 7.8–45.9%, *P* = .006), and there was slight heterogeneity among the 8 studies (*I*^2^ = 56.4%, *P* = .025). Subgroup analyses were conducted for different factors, including the type of cancer (HCC or other cancers), therapeutic regimen (RITC or RIT), and country (China or USA), and the pooled rates are shown in Figure 8. Nearly 12 AEs were reported in the included trials (Table 3).

**Table 3:** Pooled rates of AEs in patients with solid cancers treated with RIT-base regimens.

Adverse events	Effect size (%)	95% CI (%)	*P* value	*I*^2^ (%), *P* value	Reference
All AEs	86	77–93	.00	17.29, .3	^[20–22]^
Grade 3–4 AEs	26.9	7.8–45.9	.006	56.4, .025	^[13–18,20,21]^
HCC group	8	−0.09–0.24	.381	0.0, .951	^[13,16,18,20,21]^
Other caners group	53.2	35.7–70.7	.00	0.0, .417	^[14,15,17]^
RITC group	34.7	16.5–57	.00	75.6, .017	^[13,14,21]^
RIT group	25.4	10.3–40.6	.001	43.5, .132	^[15–18,20]^
China group	31.8	18.2–45.4	.00	64.3, .016	^[13–15,18,20,21]^
USA group	20.7	17.4–41.6	.128	34.6, .025	^[13,16–18,21]^
Fever	17.8	2–37.7	.079	0.00, .396	^[13,18,21]^
Skin reaction	14	9–19	.00	0.00, .5	^[13,14,16–18,20,21]^
Aspartate aminotransferase increased	18	13–25	.00	0.00, .98	^[17–20]^
Alanine aminotransferase increased	22	14–31	.00	0.00, .91	^[17,18,20]^
Anemia	13	8–19	.00	0.00, .82	^[14,18,22]^
Leukopenia	25	11.6–38.4	.00	68.6, .023	^[14,18,19,22]^
Neutropenia	21.4	7.3–35.6	.00	0.00, .695	^[14,17,18,22]^
Thrombocytopenia	23.8	10.1–37.6	.001	26.8, .243	^[13,14,17–19]^
Diarrhea	15	7–24	.00	0.00, .53	^[13,16,20,21]^
Hypertension	9	2–18	.00	52.96, .07	^[13,17,18,20,21]^
Gastrointestinal discomfort	22	10.1–33.9	.00	0.00, .802	^[13,14,16–21]^
Fatigue	23.1	7.9–38.2	.003	0.00, .55	

AEs = adverse events, CI = confidence interval, HCC = hepatocellular carcinoma, RIT = PD1/PDL1 inhibitors combined with radiotherapy and anti-angiogenic agents, RITC = RIT plus chemotherapy.

#### 3.4.4. Sensitivity analysis.

**Figure 9.**
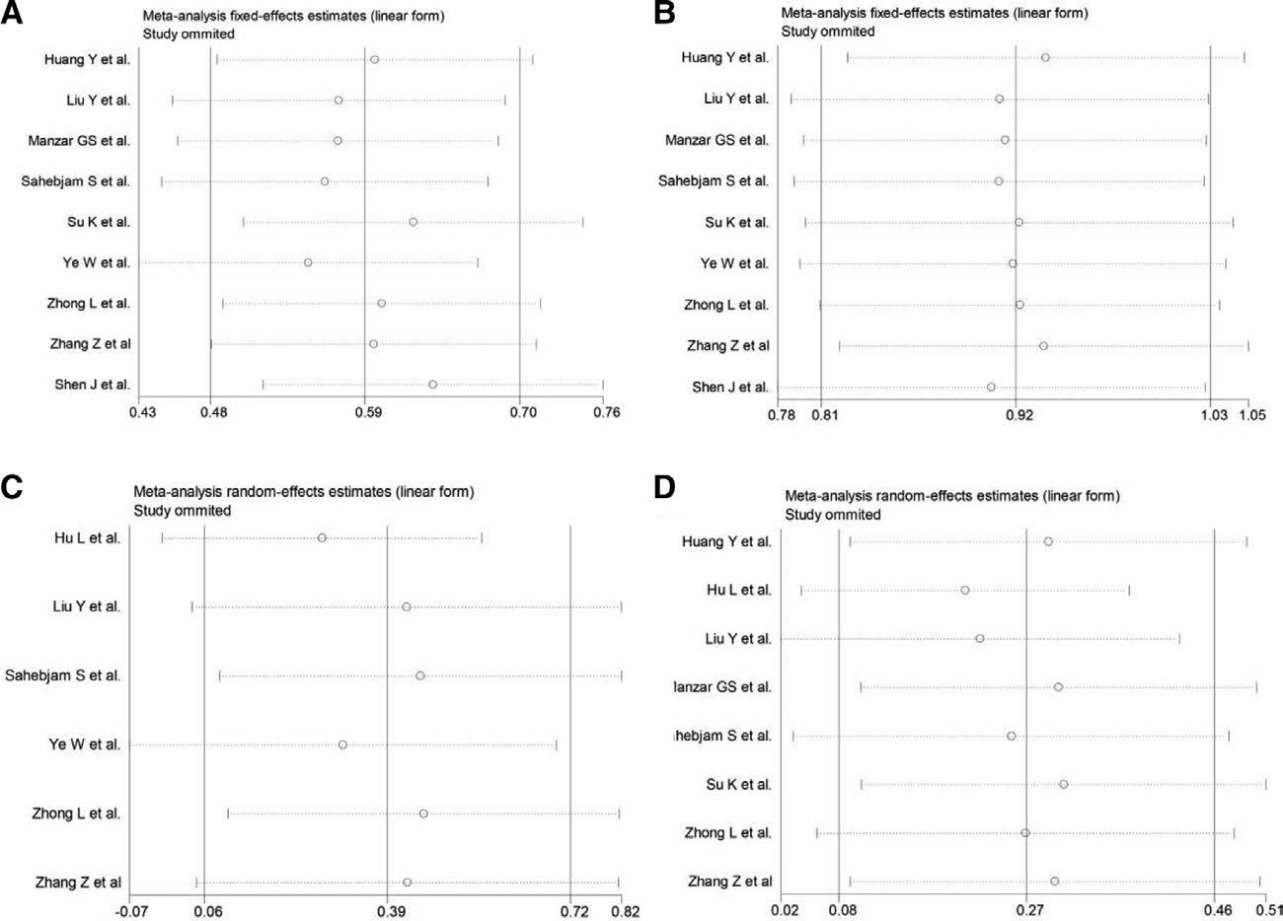
Sensitivity analysis of pooled rates for (A) ORR, (B) DCR, (C) CRR, and (D) grade over 3 AEs. AEs = adverse events, CRR = complete remission rate, DCR = disease control rate, ORR = overall response rate.

Sensitivity analysis was performed by excluding 1 individual study each time to assess the influence of each individual study on the pooled rates for ORR, DCR, CRR, and grade 3 to 4 AEs. The omission of any single study did not appreciably change the pooled rate, and the estimates in each case were well within the confidence limits of the overall estimate, indicating that our combined results were reliable (Fig. 9).

#### 3.4.5. Publication bias.

**Figure 10.**
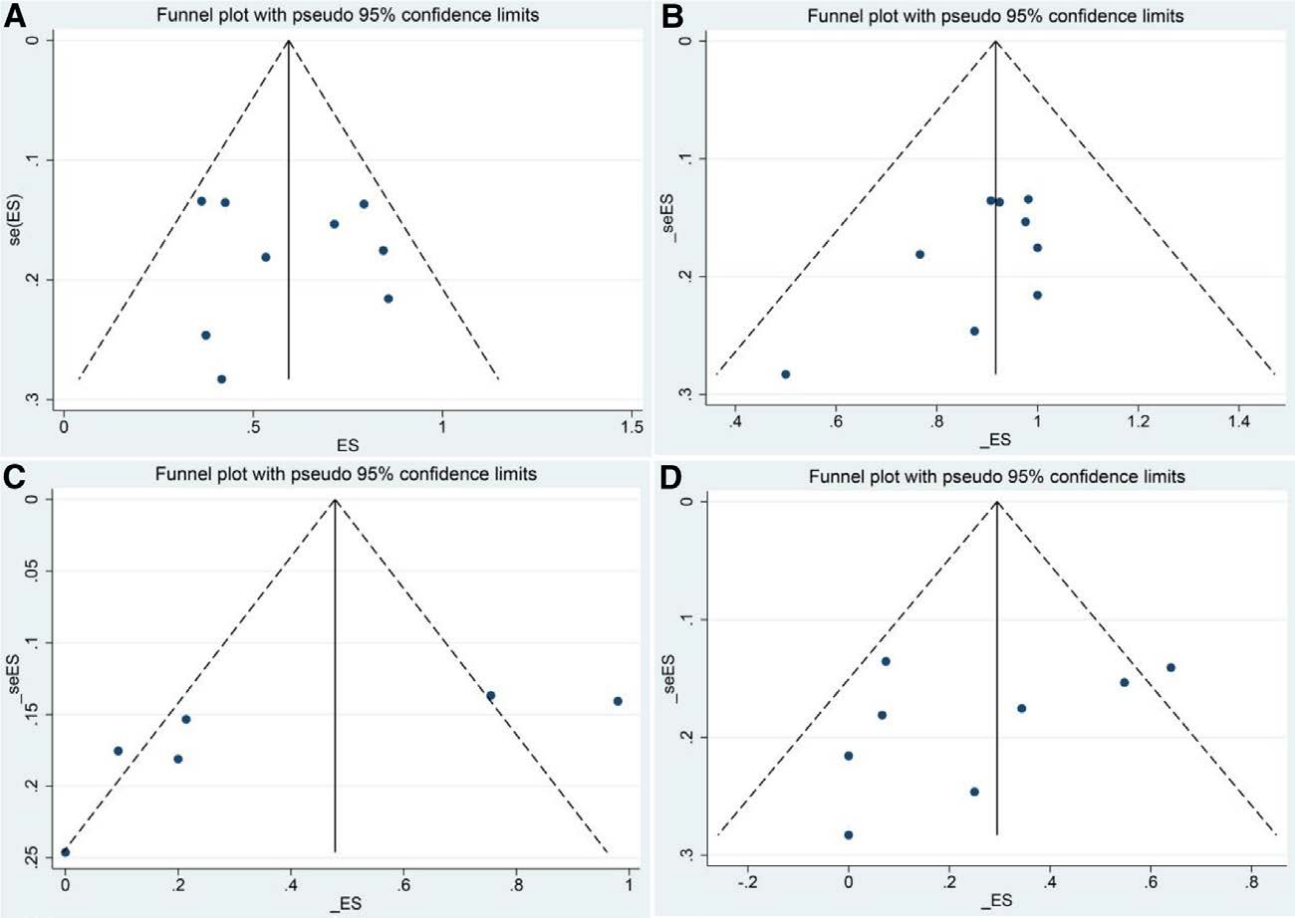
The funnel plot of the risk of bias. (A) The funnel plot of pooled ORR. (B) The funnel plot of pooled DCR. (C) The funnel plot of pooled CRR. (D) The funnel plot of grade 3 to 4 AEs. AEs = adverse events, CRR = complete remission rate, DCR = disease control rate, ORR = overall response rate, SE = standard error.

Publication bias was assessed in ten clinical trials. The funnel plot showed that the left and right sides were symmetrical (Fig. 10). Egger tests showed that *P* = .924, *P* = .113, *P* = .091, *P* = .297 for pooled ORR, DCR, CRR, grade 3 to 4 AEs, respectively. Therefore, the included studies could not be considered to have a publication bias.

## 4. Discussion

This study is the first quantitative analysis to evaluate the efficacy and safety of anti-PD1/PDL1 combined with anti-angiogenic drugs and RT. Since the majority of studies were single-arm trials, we selected tumor response and safety as the main endpoints, while extracting and analyzing data on survival from a few nonrandom comparative trials.

Overall, the pooled ORR, DCR, CRR, any-grade AEs, and grade 3 to 4 AEs of solid tumors receiving PD1/PDL1 inhibitors combined with anti-angiogenic agents and RT were 59%, 92%, 48%, 86%, and 26.9%, respectively. Nevertheless, heterogeneity caused by medication regimens, different countries, and cancer types was revealed through subgroup analyses.

We noticed that combinations of PD1/PDL1 inhibitors, RT, and anti-angiogenic drugs were mostly applied in HCC in current clinical trials. For the included solid cancers, the ORR of the patients treated with the RIT-base regiment was 59%, and the ORR of cases treated with the triple combination therapy with chemotherapy was 50%, which seemed to be worse than that without chemotherapy (62%). While there was slight heterogeneity (*I*^2^ = 47.6%, *P* = .075) in the RIT group, it may be caused by various types of cancer (4 trials on HCC, RCC, NSCLC, and a trial on all solid tumors). Furthermore, the cancer type subgroup analysis showed that the ORR of patients with HCC treated with RIT-based regimens was 51% and 65% in other cancers, while a slight heterogeneity was found in other cancer groups. The difference between HCC and others and the heterogeneity could be caused by the different sensitivity of tumors to the RIT-based regimens, which was consistent with published studies.^[23,24]^ Moreover, the ORR of patients from the USA was better than that from China (85% vs 54%) by visual inspection, which may be due to the small number of included studies from the USA; therefore, it is necessary to reanalyze as more clinical trials on the RIT regimen are published in the USA.

Additionally, our results showed that the RIT-based regimens conferred a positive efficacy for almost all included solid tumors (DCR = 92%) regardless of the medication regimens, type of cancer, and different countries. Remarkably, the CRR of patients treated with RIT-based regimens was 48%, although there was significant heterogeneity (*I*^2^ = 83.7%, *P* = .00). Therefore, subgroup analyses were conducted to examine the sources of study heterogeneity, and we found that this obvious distinction in CRR could be attributable to the treatment sensitivity of different cancers, and there was a notable difference between sensitive cancers (NPC and NSCLC) and insensitive cancers (86% vs 15%). NPC is particularly sensitive to RT,^[25,26]^ and NSCLC has a better response to targeted drugs and RT,^[27]^ while HCC, RCC, and recurrent glioma are insensitive to RT.^[28,29]^ Furthermore, the results reported that the MR of patients with HCC who received the RIT regimen was 39%, but it could be affected by many factors, including the line of therapy, age of the patient, hepatic function, and sequence of medication.^[30,31]^

In nonrandomized comparative trials, our results showed that triple combination regimens conferred an advantage in PFS and OS over monotherapy or dual-combination therapy. The OS of patients treated with monotherapy or dual-combination therapy was nearly half of those treated with RIT (*P* < .05) by univariate analysis and 0.477 (*P* < .05) by multivariable analysis. The PFS in RIT regimens was over twice that in monotherapy or dual-combination therapy, regardless of univariate or multivariate analysis.

In our study, we found that the rate of any-grade AEs was 86%, including 26.9% grade 3 to 4 AEs. We found that there was slight heterogeneity among the included trials, which could be attributed to the type of cancer through subgroup analyses. The rate of grade 3 to 4 AEs in HCC was 8%, which was lower than that in other cancers (53 %). The propensity to develop treatment-related AEs after combinational therapeutic strategies may be affected by the specific site and nature of the tumor. For example, the additive effect of hepatic injury was observed when PD1/PDL1 inhibitors were combined with RT and anti-angiogenesis agents in patients with HCC.^[32]^ Similarly, the choice of PD1/PDL1 inhibitors, tumor histology, mutational burden, and RT are associated with distinct AEs.^[33]^

Overall, the interaction between anti-PD1/PDL1, angiogenesis, and RT was thought to regulate tumor-killing immunity and tumor vasculature. Based on existing preclinical and clinical data regarding the addition of RT to the combination of PD1/PDL1 inhibitors and angiogenesis, the triple combination approach appears to be an effective treatment for solid cancers.^[34,35]^ However, triple combination therapy has long been adopted as any-line treatment in clinical practice. First, the treatment sequences, duration, and timing must be determined, as these may directly affect clinical efficacy. Moreover, based on limited data, the efficacy and safety of the triple combination remain largely unexplored and further analyses are required. Currently, there are approximately 30 any-phase trials ongoing, including KEYNOTE-B59^[36]^ and KEYNOTE-B21,^[37]^ and we expect them to have amazing results.

Our study has some limitations. First, because of the paucity of available trials in this field, the number of studies included in our analysis was low. Second, the designs of the included studies differed in several aspects, including the number of centers involved, clinical phase, duration of follow-up, and sample size. In addition, most of the studies included in the meta-analysis were single-arm clinical trials, and we could not compare the advantages and disadvantages of RIT-based, monotherapy, or dual-combination therapy. Finally, because of the different types of cancers, there was heterogeneity in the pooled effect, but the data from other cancer types, except HCC, were insufficient to conduct subgroup analysis. Finally, we expect that more clinical trials on various cancers treated with the triple combination regimen will be reported.

## 5. Conclusion

In summary, in the treatment of advanced/unresectable/metastatic solid tumors, the combination of PD1/PDL1 inhibitors, anti-angiogenic agents, and RT achieved better survival benefits than single or dual-combination therapy. In addition, combination therapy is tolerable and safe. However, randomized studies with larger patient groups are needed to confirm these results.

## Author contributions

**Conceptualization:** Feng Xian, Guohui Xu.

**Data curation:** Feng Xian, Jing Wu, Liming Zhong, Guohui Xu.

**Formal analysis:** Feng Xian, Jing Wu.

**Funding acquisition:** Guohui Xu.

**Investigation:** Feng Xian, Liming Zhong.

**Methodology:** Feng Xian, Jing Wu.

**Project administration:** Feng Xian, Jing Wu.

**Resources:** Feng Xian.

**Software:** Feng Xian, Jing Wu.

**Supervision:** Feng Xian, Guohui Xu.

**Validation:** Feng Xian.

**Visualization:** Feng Xian.

**Writing – original draft:** Feng Xian, Jing Wu.

**Writing – review & editing:** Liming Zhong, Guohui Xu.

## Supplementary Material








