# Supplementary material for: Efficacy and safety of PD1/PDL1 inhibitors combined with radiotherapy and anti-angiogenic therapy for solid tumors: A systematic review and meta-analysis
Source: Medicine (Baltimore). 2023 Mar 10;102(10):e33204. doi: 10.1097/MD.0000000000033204 (PMC9997836; doi:10.1097/MD.0000000000033204)
Supplement: Supplementary file 2 [file medi-102-e33204-s002.pdf]

Database	Search strategy
PubMed	((((((((((Tumor[Title/Abstract]) OR (Neoplasm[Title/Abstract])) OR (Neoplasia[Title/Abstract])) OR (Cancer[Title/Abstract])) OR (Malignancy[Title/Abstract])) OR (Carcinoma[Title/Abstract])) OR (Malignant Epithelial Neoplasms[Title/Abstract])) OR (Epithelioma[Title/Abstract])) OR (Carcinomatosis[Title/Abstract])) AND ((((((((((((((((((((((((((Immune Checkpoint Inhibitors[Title/Abstract]) OR (Immune Checkpoint Inhibitor[Title/Abstract])) OR (Immune Checkpoint Blockers[Title/Abstract])) OR (Immune Checkpoint Blockade[Title/Abstract])) OR (PD-L1 Inhibitors[Title/Abstract])) OR (PDL1 Inhibitors[Title/Abstract])) OR (PD-L1 Inhibitor[Title/Abstract])) OR (PDL1 Inhibitor[Title/Abstract])) OR (Programmed Death-Ligand 1 Inhibitors[Title/Abstract])) OR (Programmed Death Ligand 1 Inhibitors[Title/Abstract])) OR (PD-1-PD-L1 Blockade[Title/Abstract])) OR (PD1 PDL1 Blockade[Title/Abstract])) OR (PD-1 Inhibitors[Title/Abstract])) OR (PD1 Inhibitors[Title/Abstract])) OR (PD-1 Inhibitor[Title/Abstract])) OR (PD1 Inhibitor[Title/Abstract])) OR (Programmed Cell Death Protein 1 Inhibitor[Title/Abstract])) OR (Pembrolizumab[Title/Abstract])) OR (Nivolumab[Title/Abstract])) OR (Atezolizumab[Title/Abstract])) OR (Durvalumab[Title/Abstract])) OR (Toripalimab[Title/Abstract])) OR (Sinitilimab[Title/Abstract])) OR (Camrelizumab[Title/Abstract])) OR (Tislelizumab[Title/Abstract])) OR (Penpulimab[Title/Abstract])) OR (Zimberelimab[Title/Abstract]))) AND ((((((((((Radiotherapy[Title/Abstract]) OR (Radiotherapies[Title/Abstract])) OR (Radiation Therapy[Title/Abstract])) OR (Therapy, Radiation[Title/Abstract])) OR (Radiation Treatment[Title/Abstract])) OR (Targeted Radiotherapies[Title/Abstract])) OR (Targeted Radiotherapy[Title/Abstract])) OR (Targeted Radiation Therapy[Title/Abstract])) OR (Targeted Radiation Therapies[Title/Abstract])) OR (SBRT[Title/Abstract])) OR (Stereotactic body radiation therapy[Title/Abstract]))) AND ((((((((((((((((((((((((((((((Angiogenesis Inhibitors[Title/Abstract]) OR (Angiogenetic Antagonist[Title/Abstract])) OR (Angiogenetic Inhibitor[Title/Abstract])) OR (Angiogenic Antagonist[Title/Abstract])) OR (Angiogenic Inhibitor[Title/Abstract])) OR (Angiostatic Agent[Title/Abstract])) OR (Anti Angiogenetic Agent[Title/Abstract])) OR (Angiogenic Inhibitors[Title/Abstract])) OR (Angiostatic Agents[Title/Abstract])) OR (Anti Angiogenic Drugs[Title/Abstract])) OR (Antiangiogenic Agents[Title/Abstract])) OR (Anti Angiogenic Drug[Title/Abstract])) OR (Neovascularization Inhibitor[Title/Abstract])) OR (Antiangiogenic Agent[Title/Abstract])) OR (Angiogenesis Factor Inhibitor[Title/Abstract])) OR (Anti Angiogenesis Effect[Title/Abstract])) OR (Antiangiogenesis Effect[Title/Abstract])) OR (Anti Angiogenesis Effects[Title/Abstract])) OR (Antiangiogenic therapy[Title/Abstract])) OR (Anti-angiogenic therapy[Title/Abstract])) OR (Bevacizumab[Title/Abstract])) OR (Ramuciruma[Title/Abstract])) OR (Recombinant Human Endostatin[Title/Abstract])) OR (Endostar[Title/Abstract])) OR (Apatinib[Title/Abstract])) OR (Sorafenib[Title/Abstract])) OR (Sunitinib[Title/Abstract])) OR (Regorafenib[Title/Abstract])) OR (Lenvatinib[Title/Abstract])) OR (Anlotinib[Title/Abstract])) OR (Erdafitinib[Title/Abstract]))
Web of Science	#1. TS=(Tumor OR Neoplasm OR Neoplasia OR Cancer OR Malignancy OR Carcinoma OR Malignant Epithelial Neoplasms OR Epithelioma OR Carcinomatosis)
	#2. TS=(Immune Checkpoint Inhibitors OR Immune Checkpoint Inhibitor OR Immune Checkpoint Blockers OR Immune Checkpoint Blockade OR PD-L1 Inhibitors OR PDL1 Inhibitors OR PD-L1 Inhibitor OR PDL1 Inhibitor OR Programmed Death-Ligand 1 Inhibitors OR Programmed Death Ligand 1 Inhibitors OR PD-1-PD-L1 Blockade OR PD1 PDL1 Blockade OR PD-1 Inhibitors OR PD1 Inhibitors OR PD-1 Inhibitor OR PD1 Inhibitor OR Programmed Cell Death Protein 1 Inhibitor OR Pembrolizumab OR Nivolumab OR Atezolizumab OR Durvalumab OR Toripalimab OR Sinitilimab OR Camrelizumab OR Tislelizumab OR Penpulimab OR Zimberelimab)
	#3. TS=(Radiotherapy OR Radiotherapies OR Radiation Therapy OR Therapy, Radiation OR Radiation Treatment OR Targeted Radiotherapies OR Targeted Radiotherapy OR Targeted Radiation Therapy OR Targeted Radiation Therapies OR SBRT OR Stereotactic body radiation therapy)
	#4. TS=(Angiogenesis Inhibitors OR Angiogenetic Antagonist OR Angiogenetic Inhibitor OR Angiogenic Antagonist OR Angiogenic Inhibitor OR Angiostatic Agent OR Anti Angiogenetic Agent OR Angiogenic Inhibitors OR Angiostatic Agents OR Anti Angiogenic Drugs OR Antiangiogenic Agents OR Anti Angiogenic Drug OR Neovascularization Inhibitor OR Antiangiogenic Agent OR Angiogenesis Factor Inhibitor OR Anti Angiogenesis Effect OR Antiangiogenesis Effect OR Anti Angiogenesis Effects OR Antiangiogenic therapy OR Anti-angiogenic therapy OR Bevacizumab OR Ramuciruma OR Recombinant Human Endostatin OR Endostar OR Apatinib OR Sorafenib OR Sunitinib OR Regorafenib OR Lenvatinib OR Anlotinib OR Erdafitinib)
	#5. #1 AND #2 AND #3 AND #4
Embase	#1. 'tumor':ab,ti OR 'neoplasm':ab,ti OR 'neoplasia':ab,ti OR 'cancer':ab,ti OR 'malignancy':ab,ti OR 'carcinoma':ab,ti OR 'malignant epithelial neoplasms':ab,ti OR 'epithelioma':ab,ti OR 'carcinomatosis':ab,ti
	#2. 'immune checkpoint inhibitors':ab,ti OR 'immune checkpoint inhibitor':ab,ti OR 'immune checkpoint blockers':ab,ti OR 'immune checkpoint blockade':ab,ti OR 'pd-l1 inhibitors':ab,ti OR 'pdl1 inhibitors':ab,ti OR 'pd-l1 inhibitor':ab,ti OR 'pdl1 inhibitor':ab,ti OR 'programmed death-ligand 1 inhibitors':ab,ti OR 'programmed death ligand 1 inhibitors':ab,ti OR 'pd-1-pd-l1 blockade':ab,ti OR 'pd1 pdl1 blockade':ab,ti OR 'pd-1 inhibitors':ab,ti OR 'pd1 inhibitors':ab,ti OR 'pd-1 inhibitor':ab,ti OR 'pd1 inhibitor':ab,ti OR 'programmed cell death protein 1 inhibitor':ab,ti OR 'pembrolizumab':ab,ti OR 'nivolumab':ab,ti OR 'atezolizumab':ab,ti OR 'durvalumab':ab,ti OR 'toripalimab':ab,ti OR 'sinitilimab':ab,ti OR 'camrelizumab':ab,ti OR 'tislelizumab':ab,ti OR 'penpulimab':ab,ti OR 'zimberelimab':ab,ti
	#3. 'radiotherapy':ab,ti OR 'radiotherapies':ab,ti OR 'radiation therapy':ab,ti OR 'therapy, radiation':ab,ti OR 'radiation treatment':ab,ti OR 'targeted radiotherapies':ab,ti OR 'targeted radiotherapy':ab,ti OR 'targeted radiation therapy':ab,ti OR 'targeted radiation therapies':ab,ti OR 'sbrt':ab,ti OR 'stereotactic body radiation therapy':ab,ti
	#4. 'angiogenesis inhibitors':ab,ti OR 'angiogenetic antagonist':ab,ti OR 'angiogenetic inhibitor':ab,ti OR 'angiogenic antagonist':ab,ti OR 'angiogenic inhibitor':ab,ti OR 'angiostatic agent':ab,ti OR 'anti angiogenetic agent':ab,ti OR 'angiogenic inhibitors':ab,ti OR 'angiostatic agents':ab,ti OR 'anti angiogenic drugs':ab,ti OR 'antiangiogenic agents':ab,ti OR 'anti angiogenic drug':ab,ti OR 'neovascularization inhibitor':ab,ti OR 'antiangiogenic agent':ab,ti OR 'angiogenesis factor inhibitor':ab,ti OR 'anti angiogenesis effect':ab,ti OR 'antiangiogenesis effect':ab,ti OR 'anti angiogenesis effects':ab,ti OR 'antiangiogenic therapy':ab,ti OR 'anti-angiogenic therapy':ab,ti OR 'bevacizumab':ab,ti OR 'ramuciruma':ab,ti OR 'recombinant human endostatin':ab,ti OR 'endostar':ab,ti OR 'apatinib':ab,ti OR 'sorafenib':ab,ti OR 'sunitinib':ab,ti OR 'regorafenib':ab,ti OR 'lenvatinib':ab,ti OR 'anlotinib':ab,ti OR 'erdafitinib':ab,ti
	#5. #1 AND #2 AND #3 AND #4
Cochrane Library	#1. (Tumor):ab,ti,kw OR (Neoplasm):ab,ti,kw OR (Neoplasia):ab,ti,kw OR (Cancer):ab,ti,kw OR (Malignancy):ab,ti,kw OR (Carcinoma):ab,ti,kw OR (Malignant Epithelial Neoplasms):ab,ti,kw OR (Epithelioma):ab,ti,kw OR (Carcinomatosis):ab,ti,kw
	#2. (Immune Checkpoint Inhibitors):ab,ti,kw OR (Immune Checkpoint Inhibitor):ab,ti,kw OR (Immune Checkpoint Blockers):ab,ti,kw OR (Immune Checkpoint Blockade):ab,ti,kw OR (PD-L1 Inhibitors):ab,ti,kw OR (PDL1 Inhibitors):ab,ti,kw OR (PD-L1 Inhibitor):ab,ti,kw OR (PDL1 Inhibitor):ab,ti,kw OR (Programmed Death-Ligand 1 Inhibitors):ab,ti,kw OR (Programmed Death Ligand 1 Inhibitors):ab,ti,kw OR (PD1 PDL1 Blockade):ab,ti,kw OR (PD-1 Inhibitors):ab,ti,kw OR (PD1 Inhibitors):ab,ti,kw OR (PD-1 Inhibitor):ab,ti,kw OR (PD1 Inhibitor):ab,ti,kw OR (Programmed Cell Death Protein 1 Inhibitor):ab,ti,kw OR (Pembrolizumab):ab,ti,kw OR (Nivolumab):ab,ti,kw OR (Atezolizumab):ab,ti,kw OR (Durvalumab):ab,ti,kw OR (Toripalimab):ab,ti,kw OR (Sinitilimab):ab,ti,kw OR (Camrelizumab):ab,ti,kw OR (Tislelizumab):ab,ti,kw OR (Penpulimab):ab,ti,kw OR (Zimberelimab):ab,ti,kw
	#3. (Radiotherapy):ab,ti,kw OR (Radiotherapies):ab,ti,kw OR (Radiation Therapy):ab,ti,kw OR (Therapy, Radiation):ab,ti,kw OR (Radiation Treatment):ab,ti,kw OR (Targeted Radiotherapies):ab,ti,kw OR (Targeted Radiotherapy):ab,ti,kw OR (Targeted Radiation Therapy):ab,ti,kw OR (Targeted Radiation Therapies):ab,ti,kw OR (SBRT):ab,ti,kw OR (Stereotactic body radiation therapy):ab,ti,kw
	#4. (Angiogenesis Inhibitors):ab,ti,kw OR (Angiogenetic Antagonist):ab,ti,kw OR (Angiogenetic Inhibitor):ab,ti,kw OR (Angiogenic Antagonist):ab,ti,kw OR (Angiogenic Inhibitor):ab,ti,kw OR (Angiostatic Agent):ab,ti,kw OR (Anti Angiogenetic Agent):ab,ti,kw OR (Angiogenic Inhibitors):ab,ti,kw OR (Angiostatic Agents):ab,ti,kw OR (Anti Angiogenic Drugs):ab,ti,kw OR (Antiangiogenic Agents):ab,ti,kw OR (Anti Angiogenic Drug):ab,ti,kw OR (Neovascularization Inhibitor):ab,ti,kw OR

	(Antiangiogenic Agent):ab,ti,kw OR (Angiogenesis Factor Inhibitor):ab,ti,kw OR (Anti Angiogenesis Effect):ab,ti,kw OR (Antiangiogenesis Effect):ab,ti,kw OR (Anti Angiogenesis Effects):ab,ti,kw OR (Antiangiogenic therapy):ab,ti,kw OR (Anti-angiogenic therapy):ab,ti,kw OR (Bevacizumab):ab,ti,kw OR (Ramuciruma):ab,ti,kw OR (Recombinant Human Endostatin):ab,ti,kw OR (Endostar):ab,ti,kw OR (Apatinib):ab,ti,kw OR (Sorafenib):ab,ti,kw OR (Sunitinib):ab,ti,kw OR (Regorafenib):ab,ti,kw OR (Lenvatinib):ab,ti,kw OR (Anlotinib):ab,ti,kw OR (Erdafitinib):ab,ti,kw
	#5. #1 AND #2 AND #3 AND #4
