# Supplementary material for: Efficacy and safety of PD1/PDL1 inhibitors combined with radiotherapy and anti-angiogenic therapy for solid tumors: A systematic review and meta-analysis
Source: Medicine (Baltimore). 2023 Mar 10;102(10):e33204. doi: 10.1097/MD.0000000000033204 (PMC9997836; doi:10.1097/MD.0000000000033204)

Supplement Figure 1 The pooled CR in subgroup by country and therapeutic regimen.

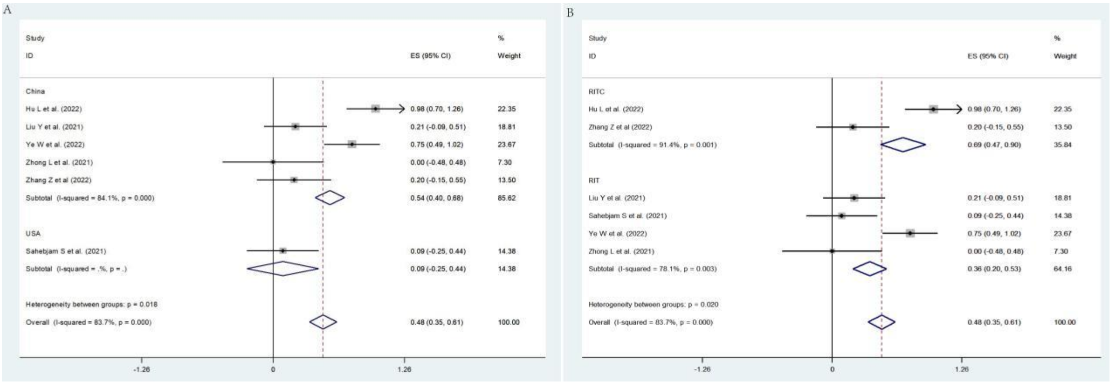

Supplement Figure 2 Sensitivity analysis of the six trials for CR pooled.

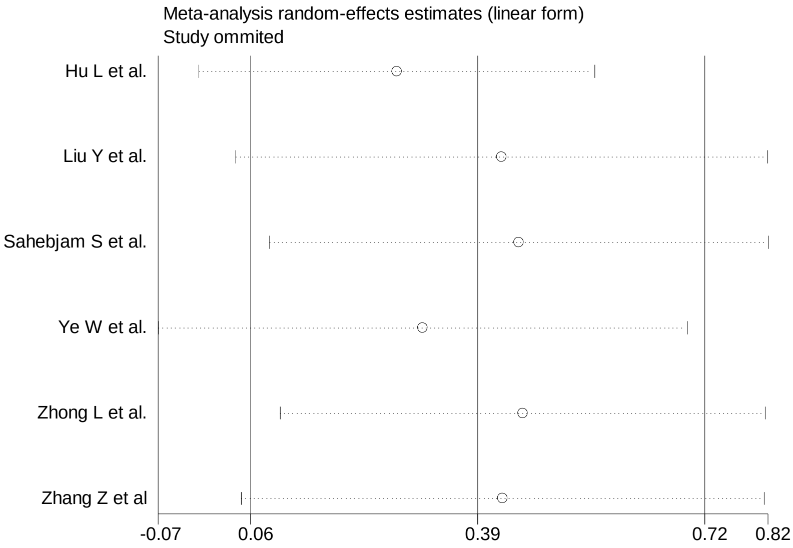

Supplement Figure 3 Galbraith plot for heterogeneity of CR pooled.

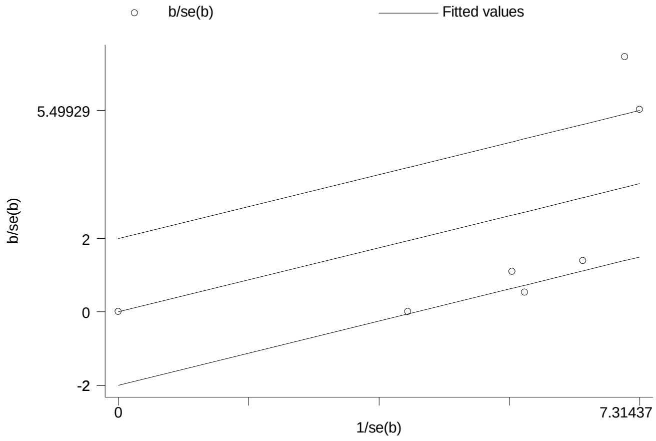

Supplement: Supplementary file 3 [file medi-102-e33204-s003.pdf]
