# Supplementary figures and images for: Efficacy and safety of PD1/PDL1 inhibitors combined with radiotherapy and anti-angiogenic therapy for solid tumors: A systematic review and meta-analysis
Source: Medicine (Baltimore). 2023 Mar 10;102(10):e33204. doi: 10.1097/MD.0000000000033204 (PMC9997836; doi:10.1097/MD.0000000000033204)

**Supplement Figure 4** Sensitivity analysis for pooled MR(A) and funnel plot for pooled MR(B).

**A**

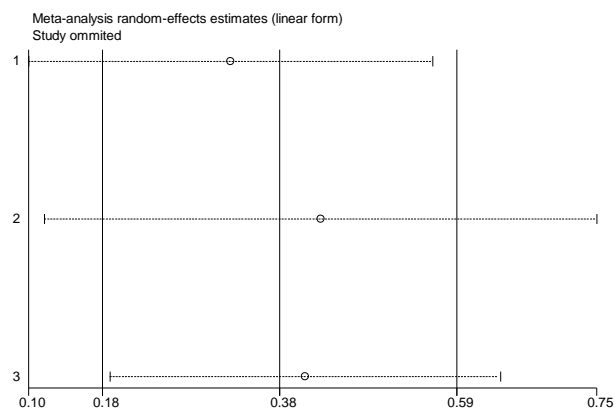

**B**

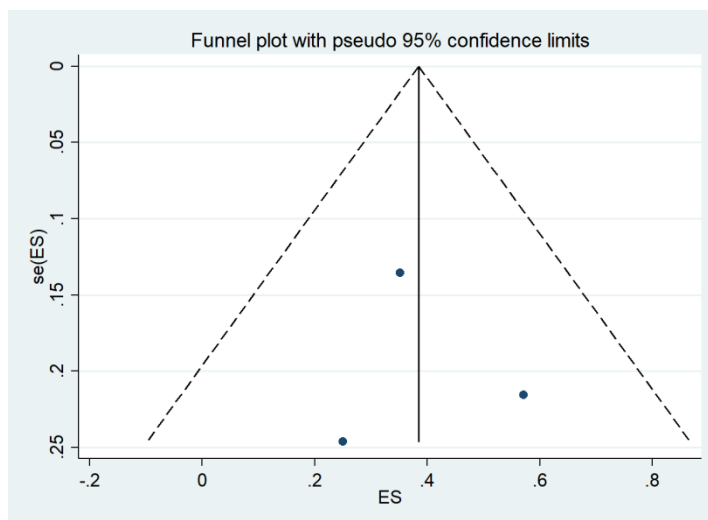

Supplement: Supplementary file 4 [file medi-102-e33204-s004.pdf]
